# NMR study of human macroPARPs domains: ^1^H, ^15^N and ^13^C resonance assignment of hPARP14 macro domain 2 in the free and the ADPr bound state

**DOI:** 10.1007/s12104-022-10110-6

**Published:** 2022-09-15

**Authors:** Nikolaos K. Fourkiotis, Periklis Charalampous, Aikaterini C. Tsika, Konstantina P. Kravvariti, Christos Sideras-Bisdekis, Angelo Gallo, Georgios A. Spyroulias

**Affiliations:** Department of Pharmacy, University of Patras, 26504 Patras, Greece

**Keywords:** HPARP14, Human macroPARPs, Macro domains, SARS-CoV-2, ADPr, Solution NMR-spectroscopy

## Abstract

hPARP14 is a human ADP-ribosyl-transferase (ART) that belongs to the macroPARPs family, together with hPARP9 and hPARP15. It contains a tandem of three macro domains (MD) while each of them has different properties. The first one, namely MD1, has not been reported to exhibit a high binding affinity for ADP-ribose (ADPr) in contrast to the following two (MD2 and MD3). All three MDs exhibit an α/β/α sandwich-like fold as reported by the deposited crystallographic structures. MD2 and MD3 recognize mono-ADP-ribosylated (MARylated) but not poly-ADP-ribosylated (PARylated) substrates and thus they allow hPARP14 to bind its targets, which can be potentially MARylated by its catalytic domain (CD). hPARP14 participates in DNA damage repair process and immune response against viruses like SARS-CoV-2, which also harbors an MD fold. Furthermore, hPARP14 like the other two macroPARPs (hPARP9 and hPARP15), is implicated in numerous types of cancer, such as B-aggressive lymphoma and sarcoma, rendering its MDs as potential important drug targets. Herein, we report the complete NMR backbone and side chain assignment (^1^H, ^13^C, ^15^N) of hPARP14 MD2 in the free and ADPr bound states and the NMR chemical shift-based prediction of its secondary structure elements. This is the first reported NMR study of a hPARP macro domain, paving the way to screen by NMR chemical compounds which may alter the ability of hPARP14 to interact with its substrates affecting its function.

## Biological context

ADP-ribosylation is a post-translational modification that plays an important role in many biological processes/pathways. Amongst them DNA damage repair and cell proliferation, being also a major “player” in stress and immune responses (Lüscher et al. 2018). It is catalyzed by enzymes called ADP-ribosyl-transferases (ARTs), which group also includes the poly(ADP)ribose polymerases (PARPs). In humans, PARPs constitute a superfamily of 17 intracellular enzymes that catalyze the addition of one or multiple ADPr moieties, using NAD^+^, on target substrates such as proteins and nucleic acids. In the first case, ADPr is transferred onto amino acid side chains with nucleophilic oxygen, nitrogen, or sulfur whereas nucleic acids are ADP-ribosylated at their phosphorylated ends (Munnur et al. 2019). The addition of one ADPr unit is referred as MARylation whereas the addition of branched or linear chains of ADPr is called PARylation (Lüscher et al. 2021). hPARPs are multidomain proteins sharing a common domain which is termed as catalytic domain (CD), usually located at their C-terminus. The additional domains (e.g., WWE, RNA recognition motif, macro domain) allow them to interact with nucleic acids, other PTMs, and various proteins in order to perform their role, diversifying their properties.

The hPARPs that contain macro domains (MD) are known as macroPARPs. Namely, hPARP9 and hPARP15 contain a tandem of two MDs, while hPARP14 a tandem of three. MDs are evolutionarily highly conserved domains present in all kingdoms of life, eukaryotic, prokaryotic organisms, and in positive sense single-stranded RNA viruses. They exhibit an α/β/α sandwich-like fold, can bind ADPr and some of them are also able to hydrolytically remove ADPr units from ADP-ribosylated substrates. For the MDs of macroPARPs, the latter property has not been defined yet. However, they are the only known proteins acting both as writers and readers (via their CD and MDs, respectively) in ADP-ribosylation (Palazzo et al. 2019). The genes that encode the three macroPARPs are located on the same chromosome, 3q21 (Aguiar et al. 2005), and the expression of hPARP9 and hPARP14 is upregulated by IFN-β and IFN-γ, thus they are interferon stimulated genes (ISG) (Fehr et al. 2020).

**Fig. 1:**
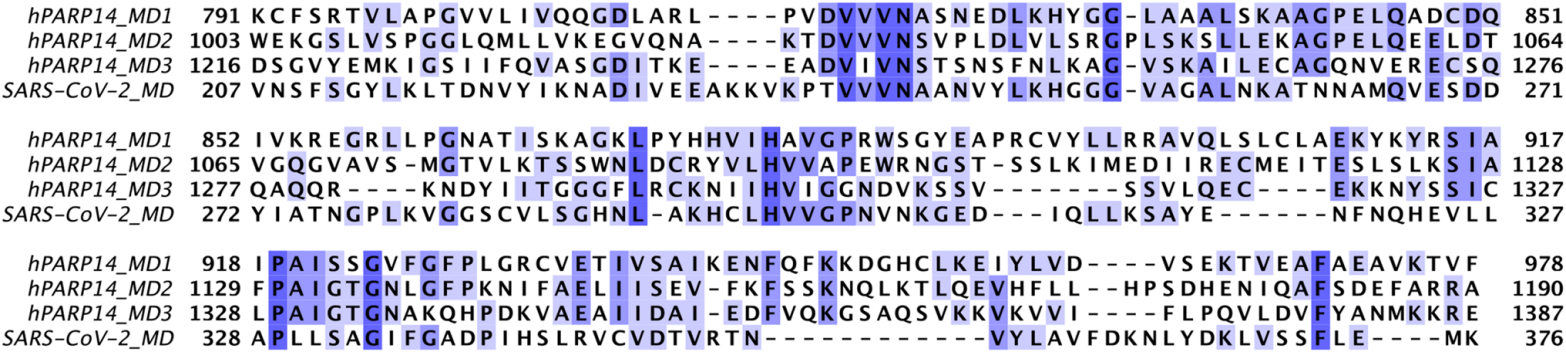
Sequence alignment of the three hPARP14 MDs and SARS-CoV-2 MD. Amino acid numbering for SARS-CoV-2 MD is presented according to the native sequence of the multidomain non-structural protein 3 (nsP3) and for hPARP14 numbering according to the native sequence of the full-length hPARP14. The color coding is dark blue for conserved residues, light blue for conserved type of residues, and white for non-conserved residues

hPARP14 (alternatively named BAL2, ARTD8, and COAST6) consists of two RNA recognition motifs (RRMs), three MDs, a WWE domain, and a CD (Schweiker et al. 2018). In contrast to MD1, MD2 and MD3 are known to bind ADPr with great affinity and to recognize MARylated but not PARylated proteins, giving hPARP14 the ability to discriminate its substrates (Forst et al. 2013). Moreover, the sequence identity of these three MDs varies from 23 to 25%. In addition, MD1 exhibits 27% sequence identity to SARS-CoV-2 MD, whereas MD2 and MD3 exhibit 16–17% (Fig. 1). Thus, MD1 is the closest related to SARS-CoV-2 MD and this fact may also indicate a molecular mimicry (An et al. 2022). The WWE domain, named after its conserved Trp and Glu residues motif that can stabilize the protein structure, binds to ADPr derivatives, and interacts with the CD involved in the MARylation of hPARP14 itself as well as of other substrates (Iwata et al. 2016; Wigle et al. 2021). hPARP14’s RRMs are located at the N-terminus of the protein and their role is to recognize RNA molecules, as the name suggests.

Among the human macroPARPs, hPARP14 is known to be overexpressed in many types of cancer such as B-aggressive lymphoma, sarcoma, and hepatocellular carcinoma. Furthermore, it is involved in inflammation and recently has gained the attention of the scientific community due to its potential role in countering viral infections including the ongoing Covid-19 pandemic. hPARP14 acts against SARS-CoV-2 by stimulating and regulating the IFN-I response via MARylating key viral and host proteins and thus activating the host defense system (Tauber et al. 2021). However, the virus can counteract the MARylating activity of hPARPs, by employing its macro domain, which in turn can remove the modification (Alhammad et al. 2021). The understanding of hPARP14 function and especially the role of each distinct domain is of high scientific importance due to their implication in many different biological pathways.

Herein, we report the almost complete NMR backbone and side chains assignments of the second MD of hPARP14 (hPARP14 MD2) in its free and ADPr bound form. Although the structure of MD2 in the ADPr bound form has already been determined via X-ray crystallography (PDB ID: 3Q71), the NMR assignment and analysis give valuable information on its dynamics and its interaction with possible ligands in a condition that approaches the natural in vivo environment.

## Methods and experiments

### Construct design

The used coding sequence of the hPARP14 MD2 corresponds to the residues 999–1191 of the full-length hPARP14 (UniProt entry: Q460N5). The gene corresponding to hPARP14 MD2 was amplified from a synthetic and codon optimized for *Escherichia coli* expression gene purchased from Twist Biosciences and was cloned into a pETM-41 vector.

The primers’ sequences used are: forward 5′-CATGCCATGGGAGGTAAGACATC-3′ and reverse 5′-ATAGTTTAGCGGCCGCTTATTAATTTGCGCGAC-3′. The obtained construct was verified by DNA sequencing. The produced polypeptide contained an N-terminal His_6_-MBP-tag and a tobacco etch virus (TEV) cleavage site, while the final derived and studied molecule contained four artificial N-terminal residues (GAMG).

### Protein expression and uniform ^15^N and ^15^N/^13^C labeling

For the expression and purification of hPARP14 MD2, the plasmid encoding the gene was used to transform Rosetta™ 2(DE3) pLysS. An LB pre-culture was inoculated with the cells and was incubated at 37 °C at 180 rpm for 14–16 h. Α culture of 0.5 L M9 medium (40 mM Na_2_HPO_4_, 22 mM KH_2_PO_4_, 8 mM NaCl) containing 0.5 g ^15^N labeled NH_4_Cl and 2 g unlabeled or ^13^C d-glucose, 1 mL from a stock solution containing 0.5 mg/mL biotin and 0.5 mg/mL thiamine, 0.5 mL of 1 M Mg_2_SO_4_, 0.15 mL of 1 M CaCl_2_, 1 mL of solution Q (40 mM HCl, 50 mg/L FeCl_2_·4H_2_O, 184 mg/L CaCl_2_·2H_2_O, 64 mg/L H_3_BO_3_, 18 mg/L CoCl_2_·6H_2_O, 4 mg/L CuCl_2_·2H_2_O, 340 mg/L ZnCl_2_, 710 mg/L Na_2_MoO_4_·2H_2_O, 40 mg/L MnCl_2_·4H_2_O), and antibiotics (kanamycin and chloramphenicol) at the appropriate concentrations, was inoculated with the preculture. The cells were incubated at 37 °C at 180 rpm and the expression was induced at O.D. value 0.6–0.8 by 1 mM IPTG lowering the temperature at 18 °C. After 14–16 h the cells were harvested.

### Protein purification and sample preparation

The protein purification protocol is reported elsewhere (Tsika et al. 2022). Protein NMR samples in the free and ADPr bound form of hPARP14 MD2 were characterized in buffers containing: 50 mM HEPES pH 7.0, 100 mM NaCl for the free form and 10 mM HEPES pH 7.0, 20 mM NaCl for the ADPr bound form. Different buffer conditions were required between the two forms due to stability reasons. Both samples contained as additives 2 mM DTT, 2 mM EDTA, 10% D_2_O, 2 mM NaN_3_, protease inhibitor cocktail (Sigma Aldrich® P8849) and 0.25 mM DSS (4,4-dimethyl-4-silapentane-1-sulfonic acid) as internal ^1^H chemical shift standard. ^13^C and ^15^N chemical shifts were referenced indirectly to the ^1^H standard using a conversion factor derived from the ratio of NMR frequencies (Wishart et al. 1995). The concentrations of NMR samples were: 0.6 mM for hPARP14 MD2 in the free form and 0.7 mM for hPARP14 MD2 in the ADPr bound form (molar ratio hPARP14 MD2:ADPr - 1:5).

### Data acquisition, processing, and assignments

All NMR experiments were recorded at 298 K on a Bruker Avance III High-Definition four-channel 700 MHz NMR spectrometer equipped with a cryogenically cooled 5 mm ^1^H/^13^C/^15^N/D Z-gradient probe (TCI). The NMR experiments used for backbone and side chains assignment are summarized in Table 1. Resonances assignment for hPARP14 MD2 in the free and in the ADPr bound form was achieved analyzing the following series of heteronuclear experiments: 2D [^1^H,^15^N]–HSQC and 2D [^1^H,^15^N]–TROSY, 3D HN(CO)CA, 3D HNCA, 3D TROSY HN(CO)CACB, 3D TROSY HNCACB, 3D HN(CA)CO, 3D HNCO, 3D HBHA(CO)NH, and hCCH–TOCSY (Table 1). All NMR spectra were processed with TOPSPIN 4.1.1 and analyzed using CARA 1.9.2a4 (Keller 2004).

**Table 1:** List of NMR experiments acquired, including the main parameters used, to perform the sequence specific assignment of the hPARP14 MD2 in the free and ADPr bound form

	Time domain data size (points)			Spectral width (ppm)			ns	Delay time (s)
	t1	t2	t3	F1	F2	F3		
^1^H–^15^N HSQC	512	2048		44.0 (^15^N)	16.0 (^1^H)		8	1.0
^1^H–^15^N TROSY	512	2048		40.0 (^15^N)	14.0 (^1^H)		2	1.0
TROSY-HN(CO)CACB	96	40	1024	72.0 (^15^N)	44.0 (^15^N)	14.0 (^1^H)	16	1.0
TROSY-HNCACB	96	40	1024	72.0 (^15^N)	44.0 (^15^N)	14.0 (^1^H)	16	1.0
HN(CA)CO	64	40	1024	18.0 (^13^C)	44.0 (^15^N)	14.0 (^1^H)	8	1.0
HNCO	64	40	1024	18.0 (^13^C)	44.0 (^15^N)	14.0 (^1^H)	8	1.0
HNCA	80	40	1024	42.0 (^13^C)	44.0 (^15^N)	14.0 (^1^H)	8	1.0
HN(CO)CA	80	40	1024	42.0 (^13^C)	44.0 (^15^N)	14.0 (^1^H)	8	1.0
HBHA(CO)NH	112	40	1024	8.0 (^1^H)	44.0 (^15^N)	14.0 (^1^H)	8	1.0
hCCH–TOCSY	128	48	1024	80.0 (^13^C)	80.0 (^13^C)	14.0 (^1^H)	16	1.0

All the experiments were acquired at the 700 MHz magnet

## Results

### Extent of assignments and data deposition

**Fig. 2:**
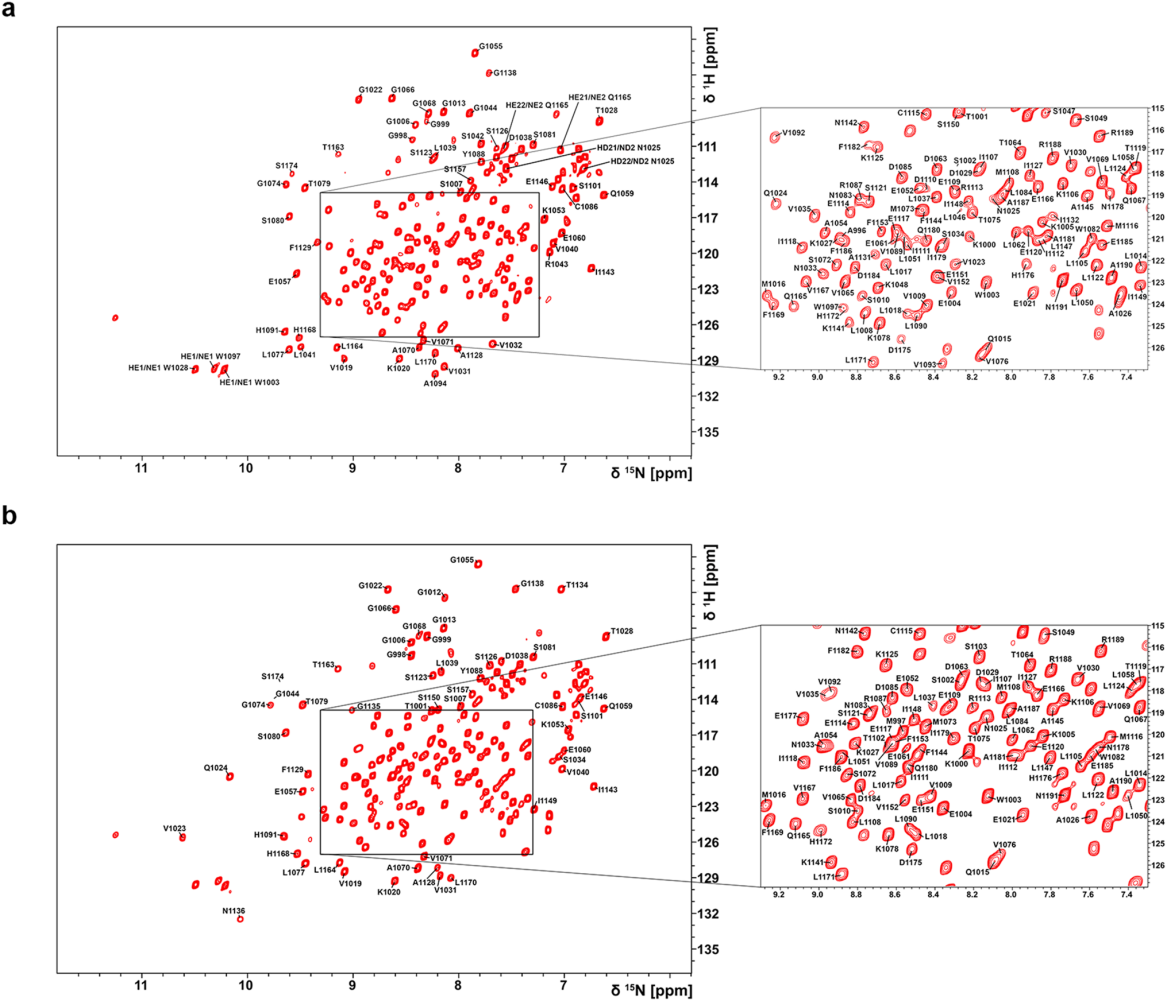
700 MHz ^1^H, ^15^N-HSQC assigned spectrum of the **a** 0.6 mM ^13^C, ^15^N-hPARP14 MD2 in the free state and **b** 0.7 mM ^13^C, ^15^N-hPARP14 MD2 with presence of ADPr (molar ratio hPARP14 MD2:ADPr - 1:5) in 50 mM HEPES pH 7.0, 100 mM NaCl, 2 mM DTT, 2 mM EDTA, 0.25 mM DSS, 10% D_2_O and 10 mM HEPES pH 7.0, 20 mM NaCl, 2 mM DTT, 2 mM EDTA, 0.25 mM DSS and 10% D_2_O respectively, acquired at 298 K. Reported amino acids are numbered according to the native sequence of the full-length hPARP14

The ^1^H, ^15^N-HSQC spectra on hPARP14 MD2 (residues 999–1191 of the full-length protein) shows a great resonance dispersion (HNs) for both forms of the protein as shown in Fig. 2a (free) and Fig. 2b (ADPr bound).

**Fig. 3:**
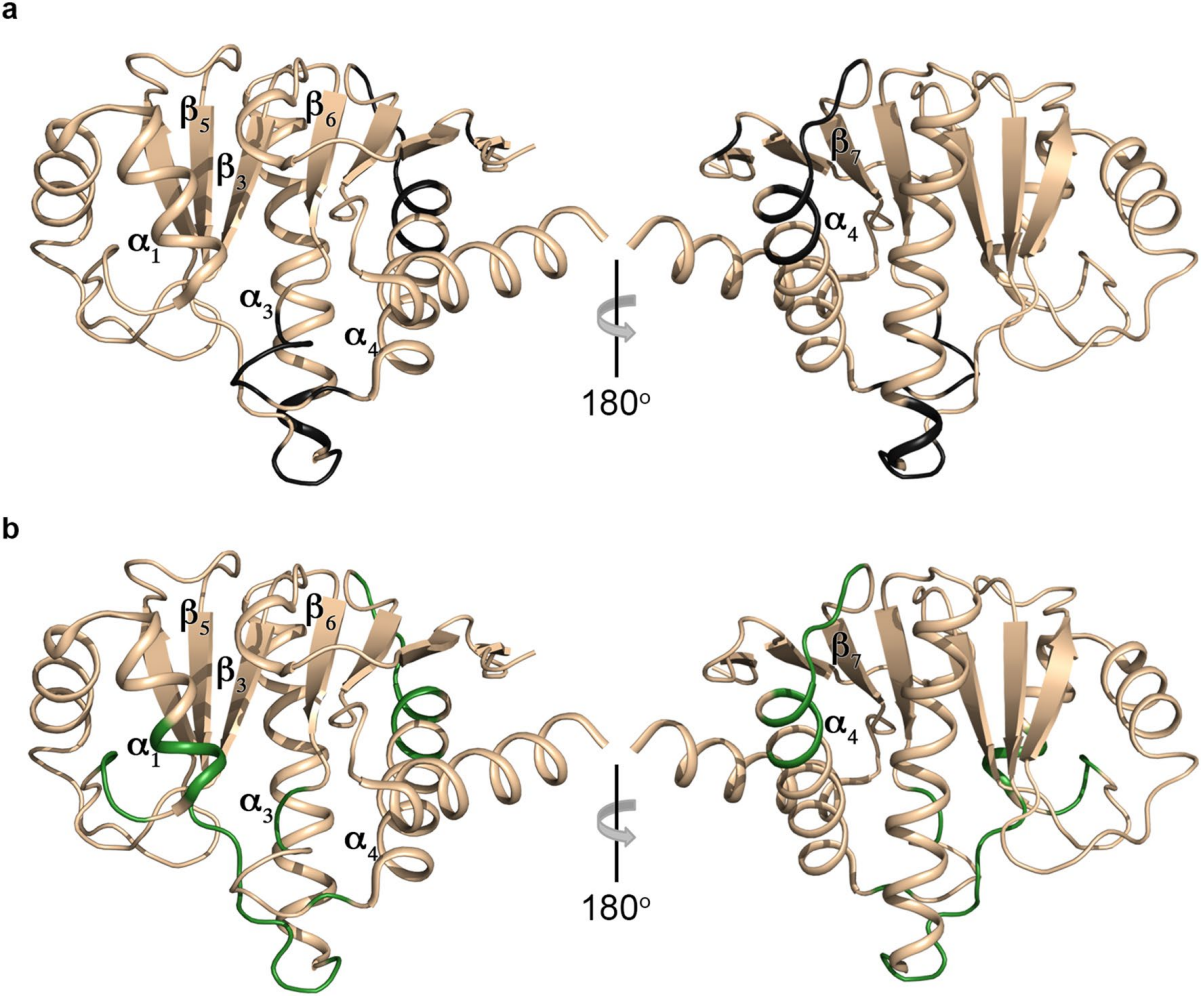
Cartoon representation of hPARP14 MD2 (PDB ID: 3VFQ). **a** Missing residues in the free form of the protein in the current study are colored in dark grey and **b** missing residues in the ADPr bound form of the protein in the current study are colored in green

For the free form of hPARP14 MD2 were assigned 166 out of 185 HNs present in the sequence (excluding from the 193 total residues the eight proline), 129 out 193 CO (backbone CO), 181 out 193 Cα and 167 out 179 Cβ. By contrast for the side chain resonances, were identified 1015 out of 1493 atoms available for the free protein using the hCCH–TOCSY experiment. The unassigned HN resonances of hPARP14 MD2 belong to G1012, R1098-G1100, T1102-S1104, G1133-L1137, F1139, L1154-S1156, K1158-K1162 and the N of all the prolines. All the assignment procedure was also repeated for the ADPr bound form, for comparison to the free form. In this case, were assigned 160 out of 185 HNs present in the hPARP14 MD2 sequence (excluding the eight proline residues), 155 out 193 CO (backbone CO), 176 out 193 Cα and 161 out 179 Cβ. By contrast for the side chain resonances, were assigned 953 out of 1493 atoms available for the protein in the ADPr bound form using the hCCH–TOCSY experiment. Specifically, for the residues L1041-R1043, P1045-K1048, V1093-G1100, I1132-G1133, F1139, L1154-S1156, K1158-K1162, was not possible to detect and assign any signals. Most of these missing residues in the free form are located in the loop connecting the structural elements β_5_-α_3_, which is the phosphate groups coordination site, and they were assigned only in the ADPr bound form (Fig. 3a). On the other hand, the amino acids of the β_6_-α_4_ loop, which is close in space to the β_5_-α_3_ loop, and of the region between the β_3_-α_1_ (including the N-terminus of α1 helix) were not identified in presence of ADPr (Fig. 3b). To be noticed that the β_6_-α_4_ loop is not directly involved in the ADPr binding, whereas the β_3_-α_1_ region binds the distal ribose of the ADPr. Interestingly, the residues spanning the loop α_4_-β_7_, in the opposite side of the ADPr binding cavity, remained unassigned in both forms of hPARP14 MD2. The disappearance of the above-mentioned resonances might suggest an interesting mobility of these regions that leads to a large conformational variability between the two hPARP14 MD2 states. This dynamic range of flexibility might be the cause of the hampering of the detection of the amino acids belonging to these regions. Similar phenomena have been reported also in studies of various viral MDs (Melekis et al. 2015; Makrynitsa et al. 2015; Cantini et al. 2020; Tsika et al. 2022).

**Fig. 4:**
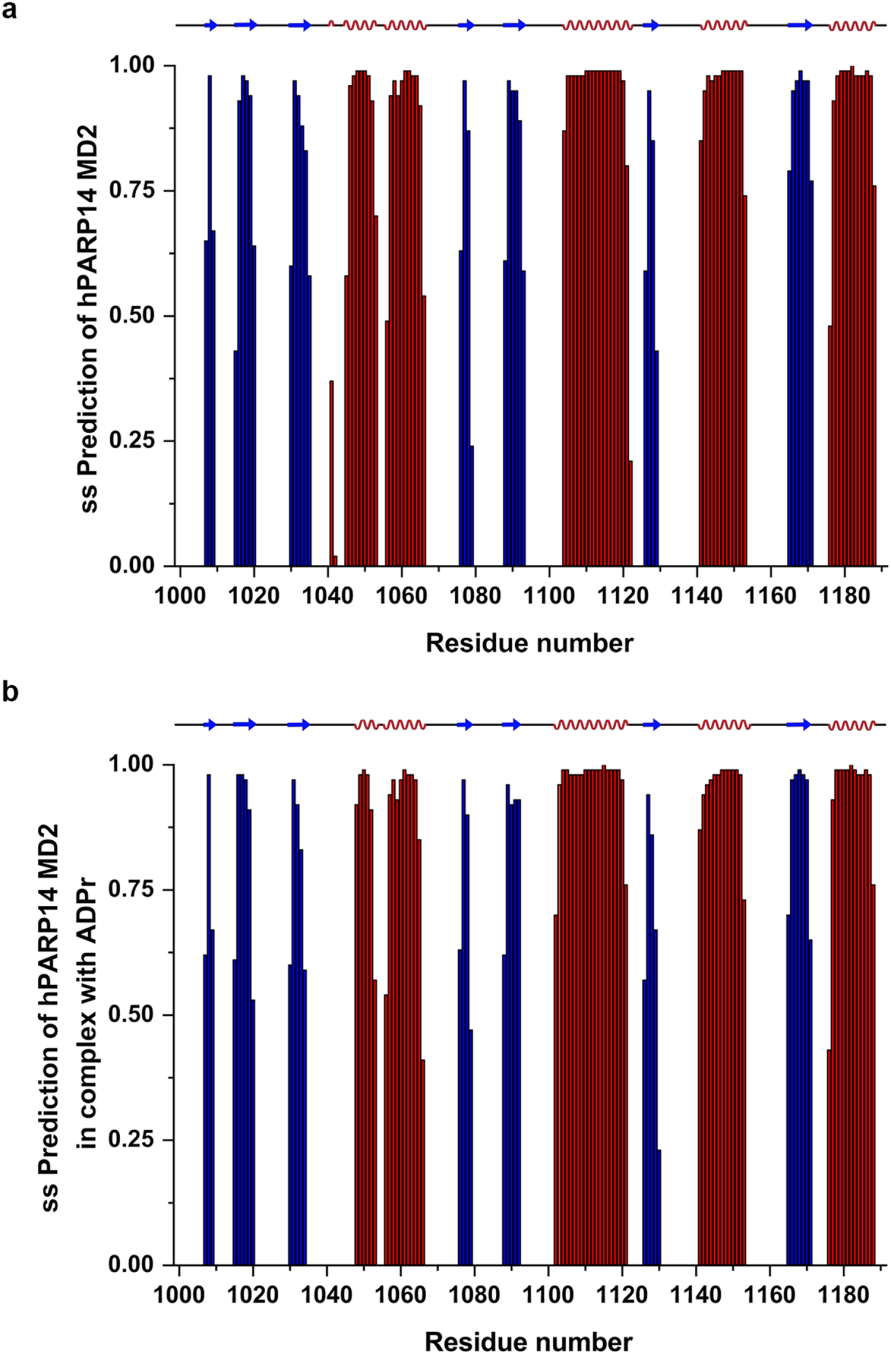
Predicted secondary structure using TALOS+ of **a** hPARP14 MD2 and **b** hPARP14 MD2 with ADPr (molar ratio hPARP14 MD2:ADPr - 1:5). Color coding red for α-helix and blue for β-sheets

Secondary structure prediction for hPARP14 MD2 in its free and ADPr bound form has been obtained, by using chemical shift assignments of five atoms (HN, Hα, Cα, Cβ, CO, N) for each residue in the sequence, by running the TALOS+ software (Shen et al. 2009). The secondary structure elements of the free hPARP14 MD2 (193 residues) show an α/β/α sandwich-like fold as follows from N- to C-terminal residues of the native sequence: β/β/β/α/α/β/β/α/β/α/β/α (Fig. 4a). Moreover, upon interaction with ADPr no significant changes in secondary structure elements are detectable (Fig. 4b).

The overall structure of hPARP14 MD2, calculated using the chemical shifts assigned and the spatial location of all the secondary structure elements, corresponds very similarly to that of the other human and viral MDs (some of them have though six instead of seven β-strands, e.g., PDB IDs 5IQ5 and 7P27). Indeed, hPARP14 MD2 has a high degree of similarity of secondary structure identity in comparison with other human MDs and even with viral MDs (Lykouras et al. 2018; Tsika et al. 2019; Makrynitsa et al. 2019). The dihedral angles predicted by TALOS+, and so the 3D structure, for free hPARP14 MD2 and its respective ADPr bound forms are in excellent agreement with the secondary structure elements found in the ADPr bound (PDB ID: 3VFQ) crystal structures. This implies that ligand binding does not alter significantly the overall secondary structure within the MDs.

Chemical shift values for the ^1^H, ^13^C, and ^15^N resonances of hPARP14 MD2 in the free state and the ADPr bound state have been deposited at the BioMagResBank (https://www.bmrb.wisc.edu) under accession numbers 51398 and 51399, respectively.

## Data Availability

Chemical shift values for the ^1^H, ^13^C, and ^15^N resonances of hPARP14 MD2 in the free state and the ADPr bound state have been deposited at the BioMagResBank (https://www.bmrb.wisc.edu) under Accession Numbers 51398 and 51399, respectively.
